# A Planned Multidisciplinary Surgical Approach to Treat Primary Pelvic Malignancies

**DOI:** 10.3390/curroncol30010084

**Published:** 2023-01-12

**Authors:** Andrea Sambri, Michele Fiore, Matteo Rottoli, Giuseppe Bianchi, Marco Pignatti, Marta Bortoli, Amelio Ercolino, Stefano Ancetti, Anna Myriam Perrone, Pierandrea De Iaco, Riccardo Cipriani, Eugenio Brunocilla, Davide Maria Donati, Mauro Gargiulo, Gilberto Poggioli, Massimiliano De Paolis

**Affiliations:** 1 Orthopedic and Traumatology Unit, IRCCS Azienda Ospedaliero-Universitaria di Bologna, 40138 Bologna, Italy; 2 General Surgery Unit, IRCCS Azienda Ospedaliero-Universitaria di Bologna, 40138 Bologna, Italy; 3 IRCCS Istituto Ortopedico Rizzoli, 40136 Bologna, Italy; 4 Plastic Surgery Unit, IRCCS Azienda Ospedaliero-Universitaria di Bologna, 40138 Bologna, Italy; 5 Division of Urology, IRCCS Azienda Ospedaliero-Universitaria di Bologna, 40138 Bologna, Italy; 6 Vascular Surgery Unit, IRCCS Azienda Ospedaliero-Universitaria di Bologna, 40138 Bologna, Italy; 7 Gynecologic Oncoloy Unit, IRCCS Azienda Ospedaliero-Universitaria di Bologna, 40138 Bologna, Italy

**Keywords:** sarcoma, pelvis, multidisciplinary, orthopedic, vascular, plastic, urology

## Abstract

The pelvic anatomy poses great challenges to orthopedic surgeons. Sarcomas are often large in size and typically enclosed in the narrow confines of the pelvis with the close proximity of vital structures. The aim of this study is to report a systematic planned multidisciplinary surgical approach to treat pelvic sarcomas. Seventeen patients affected by bone and soft tissue sarcomas of the pelvis, treated using a planned multidisciplinary surgical approach, combining the expertise of orthopedic oncology and other surgeons (colleagues from urology, vascular surgery, abdominal surgery, gynecology and plastic surgery), were included. Seven patients were treated with hindquarter amputation; 10 patients underwent excision of the tumor. Reconstruction of bone defects was conducted in six patients with a custom-made 3D-printed pelvic prosthesis. Thirteen patients experienced at least one complication. Well-organized multidisciplinary collaborations between each subspecialty are the cornerstone for the management of patients affected by pelvic sarcomas, which should be conducted in specialized centers. A multidisciplinary surgical approach is of paramount importance in order to obtain the best successful surgical results and adequate margins for achieving acceptable outcomes.

## 1. Introduction

Less than 5% of all sarcomas are located in the pelvic region. They often remain asymptomatic until they have grown large and extensively [1]. Involvement of the pelvis is one of the most unfavorable prognostic factors for bone and soft tissue sarcomas [2,3].

Complete surgical resection is the cornerstone of integrated multimodal curative treatment [4,5]. However, the pelvic anatomy is complex and poses great challenges to orthopedic surgeons, as sarcomas are often large in size. They are typically enclosed in the narrow confines of the pelvis with the close proximity of vital structures such as iliac vessels, ureters, urinary bladder and rectum [2,6,7,8,9]. Advances in perioperative radiological assessment and in surgical techniques make the aggressive management of primary pelvic sarcoma possible. Preoperative magnetic resonance imaging (MRI) allows for more accurate operative planning, thus allowing surgeons to perform more complex resections, with bone, muscular and neurovascular dissection and resection [10]. Nonetheless, the proximity of all pelvic organs makes them susceptible to injury during pelvic surgery [6,11]. Iatrogenic injuries are currently the most common cause of visceral trauma [12].

Pre-operative knowledge or suspicion of pelvic structures’ involvement may change the surgical approach or prompt the surgeon to involve other specialists such as vascular surgeons, urologists, gynecologists, plastic surgeons, and abdominal surgeons [13]. Moreover, plastic reconstruction is often needed to fill in dead space, provide support to pelvic organs, and introduce vascularized tissue for wound healing [14].

The aim of this study is to report a systematic planned multidisciplinary surgical approach for the treatment of pelvic sarcomas.

## 2. Materials and Methods

We retrospectively studied all adult (≥18 years) patients affected by bone and soft tissue sarcomas located in the pelvis that were treated at our Institution using a planned multidisciplinary approach, combining the expertise of orthopedic oncology and other surgeons (colleagues from urology, vascular surgery, abdominal surgery, gynecology and plastic surgery) during the period between January 2015 and March 2021.

Patients with chordoma were excluded because chordoma has unique clinicopathologic characteristics. Patients were excluded if surgeons were on call but never scrubbed-in, or if they were called in emergency for intraoperative complications.

The above exclusions left us with 17 patients (11 male and 6 female patients; mean age 54 years; age range, 21–74 years) who underwent combined multidisciplinary surgery for en-bloc tumor resection (Table 1).

All patients were affected by primary sarcoma. Pre-operatively, all patients were assessed with computerized tomography (CT) of the primary lesions and lung and MRI of the primary lesions. Vascular anatomy and its relation to the tumor was studied in all patients with CT angiography, to plan an adequate dissection or possible reconstruction.

**Figure 1:**
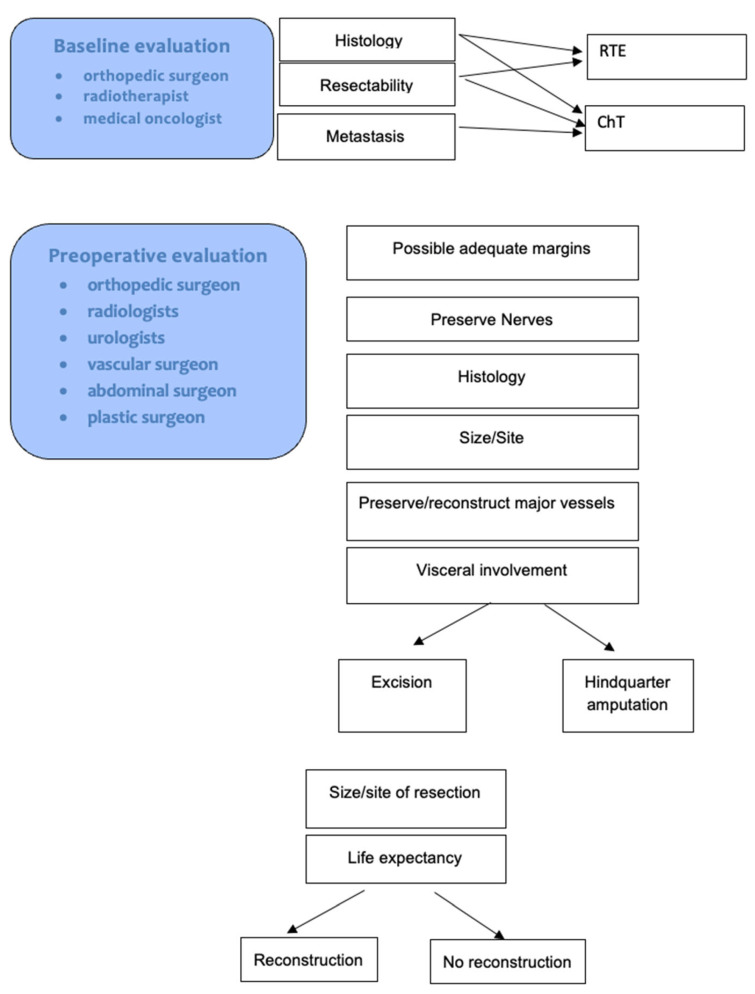
Flow diagram showing the baseline and preoperative multidisciplinary evaluations.

The use of radiotherapy (RT) and chemotherapy (ChT) was decided at the discretion of a multidisciplinary team, composed by the orthopedic surgeon, radiotherapist, and oncologist, according to soft tissue sarcomas guidelines [15] (Figure 1).

In the case of osteosarcoma, patients received ChT according to the EURAMOS protocol [16,17]. Preoperative RT was given 6–8 weeks prior to surgery in case of radio-sensitive, marginally resectable tumors. The dose of neo-adjuvant external radiotherapy was 54 Gy. In the case of histologies resistant to both radiotherapy and chemotherapy, surgery should be considered at first. This is particularly true in case of tumors that (because of site and size) are extremely hard to be operated. In case of enlargement of the tumor during neoadjuvant therapies, the patient might become inoperable even with demolitive surgeries.

All patients who received neoadjuvant therapies were further discussed after neoadjuvant therapies at a multidisciplinary board, which includes orthopedic oncologist, radiologists, urologists, vascular and abdominal surgeons and plastic surgeons. The decision on whether a limb salvage was possible was mainly based on the chance of achieving a complete tumor removal with adequate margins. This was mainly decided on the histology, size and site of the tumor, the possibility to preserve the nerves, and the possibility to preserve or reconstruct major vessels.

Preoperative selective arterial embolization was conducted in six patients, on the basis of the tumor vascularization pattern on pre-operative CT angiography. Bowel preparation was conducted the evening before surgery. Ureteral stents were always inserted preoperatively for identification of the ureters during dissection.

The expertise of surgeons other than orthopedics was required either for the protection/isolation of major vessels and pelvic organs and/or reconstruction of these structures if their sacrifice was required.

The first stage involved an anterior transabdominal (pararectal in 12 cases and median in 5) approach. The descending colon and rectum were mobilized and displaced anteriorly. The iliac vessels and ureters were mobilized and protected. The presacral space was then prepared in case of sacral resections. Abdominopelvic amputation and colostomy were not routinely necessary and were considered if there was a possibility of violating the tumor margins when dissecting the rectum from the front of the sacrum or when the tumor involved the rectum.

During en bloc, the tumor resection vascular surgery included the isolation of at least one major vessel strictly related to the tumor. This could be either preserved or reconstructed with a vascular. The decision was mainly based on the proximity to the major vessels [18,19]. All the cases had only artery reconstructed with a polytetrafluoroethylene (PTFE) vascular graft.

Pelvic bone resections were classified according to Enneking and Dunham [20]: Type I (P1) involves the iliac wing, type II (P2) the periacetabular region, type III (P3) the pubic rami, and type IV (P4) involves the sacrum.

Pelvic tumor resections were conducted with the aid of patient-specific instruments (PSI). When necessary, the pelvic anatomy was reconstructed with a custom-made 3D-printed prosthesis, as a single trabecular titanium block through the deposition of layers of titanium powder melted by electron beams technology [21,22]. The design of the prosthesis varied according to the area to be restored. The prosthesis had external small plates to allow fixation to the host bone. In the case of P2 resections, the prosthesis also had an iliac stem. The fixation on the pubic region was achieved with either a screw from the internal part of the acetabulum or a small plate. The prosthetic surface had pores with an average size of 0.7 mm, allowing the host bone to grow directly inside the implant spaces, thus increasing biological fixation. The surface of the prosthesis in contact with muscle was raw to allow a better soft tissue attachment, while the surface in contact with the abdominal viscera and vessels was smooth to reduce the risk of adherences.

Surgical margins were histologically defined according to Enneking [23]. Histologic analysis of osteosarcoma tumor map was performed in accordance with a method reported previously [24]. Patients were classified as good responders (GRs) when the percentage of tumor necrosis was 90%, when the percentage of tumor necrosis was lower, patients were defined poor responders (PRs).

After surgery, patients were followed-up with x-rays of the pelvis, CT of the pelvis and of the lungs every 3 months for the first 2 years, every 6 months for the next 3 years, and then annually. Complications were recorded [25]. Oncologic results were classified as having no evidence of disease (NED), being alive with disease because of local recurrence or metastasis (AWD) and being dead of disease (DOD).

The study is descriptive, and data are presented in total frequencies and percentages.

## 3. Results

Seven patients were treated with hindquarter amputation as the primary treatment. Ten patients underwent an excision of the tumor (Table 2).

Among five OS which received neoadjuvant ChT, two patients were GR and three were PR.

Reconstruction of bone defects after tumor resection was conducted in six patients with a custom-made 3D-printed pelvic prosthesis. Margins were wide in 12 patients and marginal in 5 patients. After the resection surgery, the plastic surgery wound coverage was necessary for three patients using a free latissimus dorsi flap.

**Table 2:** Treatment details. HA: Hindquarter amputation; ST: soft tissues.

Patient	Chemotherapy	Radiotherapy	Surgery	Additional Surgery	Multidisciplinarity	Margins	Resection	Surgery Time (Min)	Transfusion Rate (Blood Units)	Postop Length of Stay (Days)	Reconstruction
#1	Adjuvant	No	HA	Nephrectomy Abdominopelvic amputation	Urologist Abdominal surgeon Vascular surgeon	Wide	P1 + P2 + P3	298	7	21	No
#2	Adjuvant	No	HA	Nephrectomy	Urologist Vascular surgeon	Marginal	P1 + P2 + P3	203	3	15	No
#3	No	Neoadjuvant	HA	Bladder reconstruction	Urologist	Wide	P1 + P2 + P3	192	2	17	No
#4	No	Neoadjuvant	excision	Ureter reconstruction Vascular bypass	Urologist Vascular surgeon	Wide	ST excision	155	3	22	No
#5	No	No	excision	Nephrectomy Abdominopelvic amputation	Urologist Abdominal surgeon Vascular surgeon	Wide	P1	321	6	19	Custom made prosthesis
#6	No	No	excision	Vascular bypass	Vascular surgeon	Marginal	P1 + P2	237	3	16	Custom made prosthesis
#7	No	No	HA	Abdminopelvic amputation Free flap	Urologist Vascular surgeon Plastic surgeon	Wide	P1 + P2 + P3	470	7	28	No
#8	No	No	HA	Nephrectomy	Urologist Vascular surgeon	Marginal	P3	281	4	18	No
#9	Neo + adjuvant	No	HA	Cava vein trombectomy	Neurosurgery Urologist Abdominal surgeon Vascular surgeon	Wide	P1 + P2 + P3 + P4	465	16	39	No
#10	Neo + adjuvant	No	excision	Bladder reconstruction	Urologist Vascular surgeon	Wide	P1	220	5	18	Custom made prosthesis
#11	No	Neoadjuvant	excision	Vascular bypass	Vascular surgeon	Wide	ST excision	164	2	15	No
#12	No	No	excision		Urologist Vascular surgeon	Wide	P2 + P3	178	4	13	Custom made prosthesis
#13	Neo + adjuvant	No	excision	Bladder reconstruction	Urologist Abdominal surgeon Vascular surgeon	Marginal	P1	267	4	23	Custom made prosthesis
#14	No	No	HA	Free flap Nephrectomy	Urologist Abdominal surgeon Vascular surgeon Plastic surgeon	Wide	P1 + P2 + P3	521	5	31	No
#15	Neo + adjuvant	No	excision	Bladder reconstruction	Neurosurgery Urologist Abdominal surgeon Vascular surgeon	Wide	P1 + P4	240	6	33	Custom made prosthesis
#16	No	Neoadjuvant	excision	Abdominopelvic amputation Free flap	Urologist Abdominal surgeon Vascular surgeon Plastic surgeon	Wide	ST excision	558	4	63	No
#17	Neo + adjuvant	No	excision	Vascular bypass	Vascular surgeon	Marginal	P3	190	2	24	No

The mean follow-up was 32 months (range, 13–61) (Table 3). Five patients died of the disease after a mean of 48 months. Among seven patients who received ChT, three died of the disease and two are alive with disease at final follow-up.

Local recurrence was observed in 5 cases after a mean of 33 months (range, 11–41). Only one patient out of four who underwent radiotherapy developed a LR at final follow-up.

Thirteen patients experienced at least one complication. In detail, wound dehiscence was the most common complication (five patients). It was treated conservatively with wound dressing and pharmacological treatment in all cases. Three patients experienced deep vein thrombosis (DVT) treated with drugs. Two patients developed a deep seroma, which did not require surgery. In four cases, a major complication occurred, which required surgical treatment. Two patients experienced an ileo-femoral bypass occlusion during the first month after surgery, which required bypass revision. In two out of six patients treated with a custom-made prosthetic reconstruction, a periprosthetic joint infection occurred. One of these patients was effectively treated with surgical debridement, in the other case, the removal of the prosthesis and placement of cement to fill the bone defect was required.

**Table 3:** Follow up details. DOD: died of the disease; NED: no evidence of disease; AWD: alive with disease; DVT: deep venous thrombosis.

Patient	Local Recurrence	Follow Up (Months)	Status	Complications
#1	Yes	61	DOD	Wound dehiscence
#2	Yes	59	DOD	DVT
#3	No	43	NED	Wound dehiscence
#4	Yes	36	NED	Seroma DVT
#5	No	21	NED	Prosthesis infection
#6	No	49	DOD	Bypass occlusion
#7	No	55	NED	Wound dehiscence
#8	No	18	NED	Wound dehiscence DVT
#9	No	13	AWD	
#10	Yes	25	DOD	
#11	No	29	NED	Wound dehiscence
#12	No	15	NED	Seroma
#13	No	14	AWD	Bladder fistula Prosthesis infection
#14	Yes	17	NED	Wound dehiscence
#15	No	19	NED	
#16	No	45	DOD	
#17	No	33	NED	Bypass occlusion

## 4. Discussion

Patients affected by bone and soft tissue sarcomas should be treated in specialized centers, which can ensure a multidisciplinary approach based on a team composed of orthopedic oncology surgeons, vascular surgeons, plastic surgeons, urologists, and abdominal surgeons [26].

Here, we present the results for patients treated at our Institute with a planned multidisciplinary surgical approach.

The possibility of urologic, bowel, and vessels involvement in patients with pelvic malignancies should always be considered. The expertise of surgeons other than orthopedics may be required in the case of pelvic structure invasion by the tumor, for their reconstruction if their partial or complete sacrifice is necessary. Large pubic or acetabular lesions often invade the bladder and/or other urogenital organs. In addition, sarcomas frequently adhere to the peritoneum and visceral organs, in particular after neoadjuvant radiotherapy. Moreover, a multidisciplinary approach might be needed for the protection/isolation of major vessels and pelvic organs, in order to reduce the risk of complications and improve the quality of surgical margins.

Sacral and pelvic resections are often associated with complications, with series reporting rates up to 100% [27]. Urinary complications represent a major source of post-operative morbidity, most commonly from infection or urinary leak [28]. Identifying the ureters to avoid inadvertent injury is an important step in many pelvic procedures. Open-ended ureteral stents placed at the time of surgery can facilitate ureteral identification to avoid injury and, perhaps more importantly, easily identify an injury should it occur [29,30]. Ureteral injuries identified at the time of surgery are usually easily repaired whereas missed injuries can result in disastrous complications such as urinomas or urinary fistulas [31].

Vascular injuries can be predisposed by distorted anatomy and difficult perivascular tumor dissection, thus explaining the increased peri-operative vascular consultations in the setting of cancer surgery [32]. Mogannam et al. [33] reported on the role of vascular surgeons in various settings in a tertiary hospital and found that 87% were requests for intra-operative consultant assistance (26% of these because of vascular invasion and 15% for vascular exposure). In 22% of the cases the vascular surgeon was called on emergency, thus resulting in worse outcomes in total surgical time, bleeding, length of hospital stays, and post-operative vascular complications [32,33]. If the tumor invades the vascular bundle, it should not necessarily be considered as an obstacle to radical resection, but vascular surgeon intervention should be planned pre-operatively. [18,19,34] The decision to perform arterial or large vein preservation or resection may have oncological and morbid consequences, and therefore, a careful evaluation by the vascular surgery team is required [35,36].

Wounds are often unable to be closed primarily, thus requiring plastic coverage. Following adequate oncologic resection, plastic reconstruction should focus on maintaining function and aesthetic with minimal postoperative complications. This makes the plastic surgeon be actively involved in treatment planning, thus being an integral member of the multidisciplinary team [37].

At our Institution, since 2017 all complex cases of the pelvis are discussed with a multidisciplinary team. Thus, it is very hard to match this series with a similar series of patients treated without a planned multidisciplinary surgical approach. Moreover, most studies report retroperitoneal and pelvic sarcomas together as a single entity, thus making any direct comparison extremely difficult [38]. In addition, most of the previous series reporting on contiguous organ resection did not specify whether the multidisciplinary surgical approach had been planned or not [39,40,41,42]. Bonvalot et al. [43] reported on a large series of patients affected by retroperitoneal soft tissue sarcomas treated with a “frontline aggressive surgical approach”, comprising en-bloc resection of most of the adjacent uninvolved organs when in proximity of the tumor surface, while others were resected only if directly infiltrated. With this planned multidisciplinary aggressive surgery, the authors observed major complications requiring further surgery in approximately 20% of the cases. On the other hand, in a previous series on osteosarcoma of the pelvis from our Institution [44], none of the patients had a planned multidisciplinary approach. Around 35% of major complications was reported, some of which requiring a hindquarter amputation. Moreover, three patients died of surgery-related complications.

In addition to the lack of an internal control group, some further limitations study must be acknowledged. First, it is a retrospective study with possible selection biases. Moreover, the series is relatively small and heterogeneous, thus not making possible any further subgroup analysis. However, the number of samples and the heterogeneity in diagnoses are related to the rarity of individual tumors, even though our Institute is a national reference center.

## 5. Conclusions

This case series highlights that strict cooperation among surgeons is of paramount importance in all cases of complex pelvic tumor resections close to noble structures that may require intraoperative support for possible reconstruction. Well-organized collaborations between each subspecialty are the cornerstone for the management of these patients [45,46,47,48]. A multidisciplinary pre-operative evaluation is mandatory to select those cases requiring multiple specialties during surgery. Thus, an appropriate management of the patients from the diagnosis, pre-operative planning and treatment to the follow-up should be conducted in specialized centers. These should ensure a multidisciplinary approach and an extensive experience, to aim for the best successful surgical results and adequate margins achieving acceptable outcomes.

## Figures and Tables

**Table 1:** Patients’ characteristics at baseline. M: male; F: female; CS: chondrosarcoma; LS: liposarcoma; OS: osteosarcoma; LN: lymph node.

Patient	Sex	Age (Years)	Histology	Location	Metastasis at Diagnosis
#1	F	61	Myxoid Grade 2 CS	Ilium	Lungs
#2	M	74	Grade 2 CS	Ilium + acetabulum	Lungs
#3	M	66	Dedifferentiated LS	Iliac fossa	No
#4	M	66	Pleomorphic sarcoma	Iliac fossa	No
#5	M	58	Grade 3 CS	Ilium + acetabulum	No
#6	M	63	Dedifferentiated CS	Ilium + acetabulum	No
#7	M	47	Grade 2 CS	Ilium	No
#8	M	63	Grade 3 CS	Pubis	No
#9	F	21	Osteoblastic OS	Ilium + pubis + sacrum	Neoplastic thrombus
#10	F	33	Osteoblastic OS	Ilium	LN
#11	M	71	Dedifferentiated LS	Iliac fossa	No
#12	M	66	Grade 2 CS	Pubis + acetabulum	No
#13	F	32	Chondroblastic OS	Ilium	Lungs
#14	F	43	Grade 3 CS	Ilium + acetabulum	No
#15	M	61	Secondary OS	Ilium + sacrum	No
#16	F	57	Pleomorphic sarcoma	Iliac fossa	No
#17	M	41	Osteoblastic OS	Pubis	No

## Data Availability

The data presented in this study are available on request from the corresponding author.
